# The influence of chlorine in indoor swimming pools on the composition of breathing phase of professional swimmers

**DOI:** 10.1186/s12931-020-01350-y

**Published:** 2020-04-15

**Authors:** Andrzej S. Swinarew, Arkadiusz J. Stanula, Jadwiga Gabor, Paweł Raif, Jarosław Paluch, Jakub Karpiński, Klaudia Kubik, Hubert Okła, Andrzej Ostrowski, Ewaryst Tkacz, Szymon Skoczyński, Zbigniew Waśkiewicz, Thomas Rosemann, Pantelis T. Nikolaidis, Beat Knechtle

**Affiliations:** 1 Faculty of Science and Technology, University of Silesia in Katowice, 75 Pułku Piechoty 1A, 41-500 Chorzów, Poland; 2 Department of Swimming and Water Rescue, Institute of Sport Science, The Jerzy Kukuczka Academy of Physical Education, Katowice, Poland; 3 Department of Biosensors and Biomedical Signals Processing, Faculty of Biomedical Engineering, Silesian University of Technology in Gliwice, Gliwice, Poland; 4 Department of Laryngology, School of Medicine in Katowice, Medical University of Silesia in Katowice, Katowice, Poland; 5 Department of Water Sports, Academy of Physical Education, Kraków, Poland; 6 Department of Pneumonology, Faculty of Medical Sciences in Katowice, Medical University of Silesia, Katowice, Poland; 7 Department of Sports Medicine and Medical Rehabilitation, Sechenov University, Moscow, 119991 Russia; 8 Institute of Primary Care, University of Zurich, 8091 Zurich, Switzerland; 9 Exercise Physiology Laboratory, Nikaia, Greece; 10 Medbase St. Gallen Am Vadianplatz, Vadianstrasse 26, 9001 St. Gallen, Switzerland

**Keywords:** Pulmonary metabolomics, Swimming, Chloramines, Gas chromatography, Mass spectrometry, Trichloromethane, Pulmonary bioreaction

## Abstract

**Objectives:**

Swimming is one of the most popular forms of physical activity. Pool water is cleaned with chlorine, which - in combination with compounds contained in water - could form chloramines and trichloromethane in the swimmer’s lungs. The aim of the present study was to examine the effect of swimming training in an indoor pool on the composition of swimmers’ respiratory phase metabolomics, and develop a system to provide basic information about its impact on the swimmer’s airway mucosa metabolism, which could help to assess the risk of secondary respiratory tract diseases i.e. sport results, condition, and health including lung acute and chronic diseases).

**Design:**

A group of competitive swimmers participated in the study and samples of their respiratory phase before training, immediately after training, and 2 h after training were assessed.

**Methods:**

Sixteen male national and international-level competitive swimmers participated in this study. Respiratory phase analysis of the indoor swimming pool swimmers was performed. Gas chromatography combined with mass spectrometry (GCMS) was used in the measurements. All collected data were transferred to numerical analysis for trends of tracking and mapping. The breathing phase was collected on special porous material and analyzed using GCMS headspace.

**Results:**

The obtained samples of exhaled air were composed of significantly different metabolomics when compared before, during and after exercise training. This suggests that exposition to indoor chlorine causes changes in the airway mucosa.

**Conclusion:**

This phenomenon may be explained by occurrence of a chlorine-initiated bio-reaction in the swimmers’ lungs. The obtained results indicate that chromatographic exhaled gas analysis is a sensitive method of pulmonary metabolomic changes assessment. Presented analysis of swimmers exhaled air indicates, that indoor swimming may be responsible for airway irritation caused by volatile chlorine compounds and their influence on lung metabolism.

## Background

Swimming has been one of the most popular forms of physical activity, practiced by people of all ages, both for recreational and sports purposes. According to the Fitbit Activity Index [1], swimming was the third fitness activity for all ages in Great Britain, fourth in Australia, and seventh in the United States. The ability to swim has been the basis for participation in many other forms of general sport, such as sailing, wind-surfing, surfing, water polo, synchronized swimming, diving and water skiing. Swimming was also the second discipline, after athletics, in the program of the Olympic Games with the most competition, including 10 km open water race, and races at distances from 50 to 1500 m, different styles performed either individually or in relay by both sexes, resulting in a total of 34 competitions [2].

In the works devoted to swimming, it has been emphasized that this form of physical activity was one of the healthiest, providing comprehensive development of the whole organism [3]. Swimming, as any other physical human motor activity, has been associated with energy expenditure, which, depending on the type and intensity of physical exercise, resulted from various biochemical changes occurring in the human body [4, 5]. The variety of swimming competitions in terms of swimming style and race distance required a special preparation of the competitors [6]. Performing continuous work with high intensity highlighted the need of skeletal muscles for increased provision of energy substrates [7, 8].

Elite swimmers usually have trained in the pool twice a day for six days a week with 2–3 h per training session, covering a distance of 65 to 130 km weekly, depending on the style and distance [9]. During pre-race preparation, the swimmers trained mainly in indoor swimming pools where the water was treated with the help of specially developed chemical substances, most commonly sodium hypochlorite, calcium hypochlorite or ozone [10].

Chemical side products were released in swimming pools as a result of the interactions between organic matter and chlorine. They contained mainly trihalomethanes, usually represented by haloacetic acids and chloroform [11]. The chlorine present in the pool reacted with ammonia, which appeared along with urine, sweat or soap residue from users [12]. The resulting haloacetic acids contributed to the irritation of the skin and eyes. In addition, chloramines and chlorine gas irritated the respiratory system. Research has shown that problems with irritation of the nasal mucosa were very common in people regularly exercising in a swimming pool. A total of 25–74% of swimmers complained about chronic symptoms of rhinitis [13, 14]. Symptoms associated with irritation of the upper respiratory tract included nasal obstruction, pruritus, sneezing and symptoms associated with sinusitis [13–15]. In addition, swimmers often complained about a sore throat, headache and ocular symptoms [15, 16]. Current literature reports a relationship between airway dysfunction and professional swimming training [17].

It was already reported that acute exposition to chlorine may damage airways and alveoli results in acute lung injury characterized by cough and dyspnea. This prolonged exposition lead to secondary acute airway obstruction and sometimes pulmonary edema, which may convert into chronic process. The cause of chronic symptoms may be chlorine induced pulmonary inflammation and remodeling [18] sometimes called as chlorine-induced reactive airways dysfunction syndrome (RADS) [19]. Persistent chlorine induced airway irritation may be responsible for development of asthma like symptoms, but with more persistent consequences [18, 20]. Even short, but intensive exposure may result in persistent respiratory symptoms, complains and impairment of pulmonary function [21].

In acute chlorine gas exposure it is currently speculated that a transient receptor potential (TRP) channels have an important role in respiratory disease [22]. It has to be underlined that although the effect of chlorine on lungs and airways is often described as persistent in most publications acute high exposures are described [18] whereas according to our best knowledge the scarce data about effect of low dose persistent exposition on human lungs and airways. Moreover, at his stage there are no prospectively confirmed specific treatment, therefore in acute intoxication treatment regimens are symptomatic [23], however, based on experimental studies the vanilloid-type TRP channels family TRPV4 may be considered as a candidate for management of acute lung injury caused by acute chlorine gas exposure [22], but this needs to be confirmed in humans. Therefore, at this stage, asthma like syndrome caused by exposition to chlorine compounds is usually treated with anti-asthmatic drugs, but this treatment does relieve symptoms but does not ameliorate the underlying disease pathogenesis [18].

Swimmers were a specific group of athletes in whom the incidence of atopy, rhinitis, asthma and airway hyperresponsiveness was greater compared to other groups of athletes. Zwick et al. [24] noted that 79% of swimmers training 27–37 h a week reported above mentioned symptoms of upper and lower respiratory tract. Helenius et al. [25] observed swimming-induced lower airway respiratory symptoms in 57% of Finnish National Team swimmers. The most frequently reported symptoms were cough and asthma-like symptoms such as breathlessness, wheezing and chest tightness [15, 16, 26, 27]. Moreover, a large number of competitive swimmers (50–65%) were sensitized to various allergens, among which were seasonal allergens, as documented by allergy skin-prick tests, compared with a control group (29–36%) [24, 25, 28, 29]. The constant exposure of the human body to the harmful effects of chlorine by products in covered pools could cause damage to the mucosa of the airways and thus, increase nasal and lung permeability. This contributed to various types of inflammation and dangerous remodelling of the airways of highly skilled swimmers. Both upper and lower airway respiratory symptoms might have deleterious effect on swimmers’ performance and moreover, the occurring inflammation might exclude the possibility of training exercises in the water. Considering this in terms of professional swimming, it should be distinguished that even the best prepared training on land, using the most modern training devices, could not replace training in water.

Therefore, bearing in mind the fact that when a highly qualified athlete preparing to start in an important competition, he cannot afford not to participate in swimming training due to a disease resulting from the carcinogenic effect of chlorine. For this purpose, an attempt was made to investigate the effect of chlorine retention in the airways using a modern method of diagnosis of the respiratory phase [10].

At the moment, to the best of our knowledge, no research data describing the relationship between exposure to chlorine compounds and molecular chlorine, and the change in the composition of the biochemical breathing phase has been ever conducted. The change in the composition of the parity biochemical breathing phase was correlated with the long-term retention of the components of the inhaled air.

Therefore, the aim of this study was to examine the effects of swimming training in an indoor pool on the composition of swimmers’ respiratory phase. It was intended to develop a system that would provide basic information about its impact on the swimmer’s breath phase composition and metabolism. With the development of such system, it would be possible to assess the risk on the swimmers (i.e. sport results, condition, and health including lung injuries, as well as chronic diseases such as asthma in general population as well as occupational asthma in professional swimmers).

## Methods

### Ethical approval information

The study was approved by the University Bioethics Committee at the Jerzy Kukuczka Academy of Physical Education in Katowice on April 19, 2018, which consented to the conduct of research, confirmed by Resolution No. 7/2018. The subjects were informed about the purpose of the research and, written informed consent was obtained from all the participants before the commencement of the investigation.

### Subjects

The invitation to the study (inclusion criterium) was given to male competitive swimmers of ≥18 years old. The exclusion criteria included: any kind of active smoking (ex. cigarettes, IQOS) within last year or previous history of smoking (≥ 5 pack-years). Participants with any kind of chronic respiratory disease including asthma, allergic rhinitis even in cases of episodic asthma (both untreated or treated with inhaled drugs). Participants with history of tuberculosis or sarcoidosis were excluded. Assessed participants were included in their stable condition. There was a currency period lasting three months since last acute airway infection (both viral as well bacteriological). Sixteen male national and international-level competitive swimmers met inclusion criterium and were free from exclusion criteria and participated in the study. Before the commencement of the main experimental trials, participants’ physical characteristics were recorded (Table 1). Body height was assessed using a stadiometer (Seca 213, Seca GmbH & Co, Hamburg, Germany) with a precision of 0.1 cm. Body mass, percent body fat (%) and lean body mass (kg) were obtained by using the segmental multi-frequency bioimpedance analysis (InBody 720, Biospace, Seoul, South Korea) in accordance with the guidelines of the manufacturer. The maximal oxygen uptake (VO_2_max) was estimated from a maximal multistage swimming test (indirect method) [30].

**Table 1:** Physical characteristics of participants (*n* = 16)

Variables	Mean ± SD	Range	95% CI
Age (y)	20.2 ± 1.3	19–23	19.5–20.9
Mass (kg)	84.2 ± 9.2	68.5–98.0	79.3–89.1
Stature (cm)	186.1 ± 7.0	177.2–199.6	182.3–189.8
Percent body fat (%)	8.5 ± 2.8	4.5–13.8	7.0–10.0
Lean body mass (kg)	77 ± 8.2	63.5–90.6	72.6–81.3
VO_2MAX_ (ml × min^− 1^ × kg^− 1^)	60.0 ± 4.3	54.9–72.5	57.6–62.5
FINA point score (pts)	762.9 ± 70.5	660–920	722.2–803.6
Training experience (year)	13.1 ± 1.9	10–16	12.0–14.1
Volume training in week (km)	56 ± 2.9	52–60	54.5–57.5

Note: VO_2MAX_ – maximal oxygen uptake; *FINA* Fédération Internationale de Natation

During the experimental trials, the participants refrained from alcohol, caffeine and strenuous exercise for 48 h before training. The participants were fully informed of the experimental procedures and risks, and written informed consent was obtained from each participant, in accordance with the declaration of Helsinki before experiment.

### Exhaled gas analysis

**Fig. 1:**
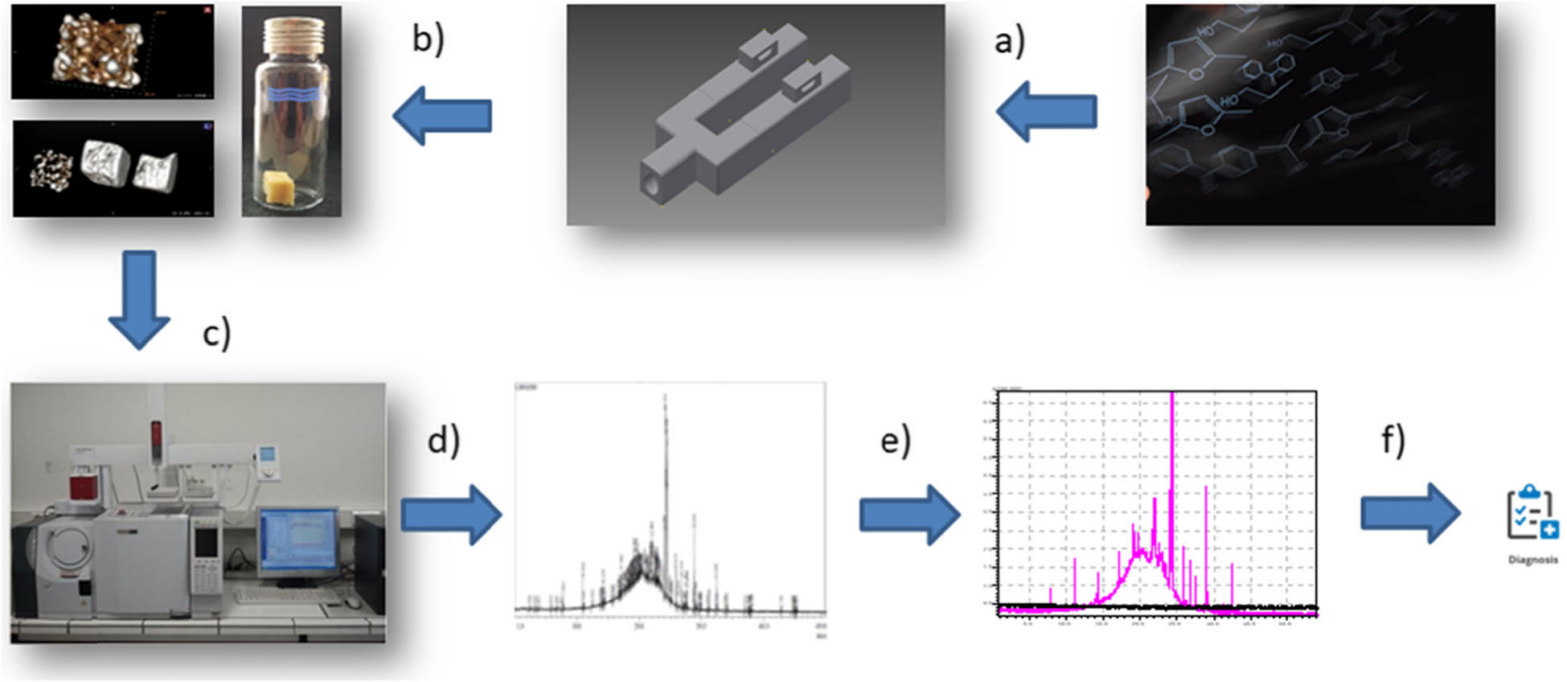
Diagram of the respiratory phase sampling procedure, **a**) adsorption to porous carbon material using a two-way patented holder, **b**) desorption of biomarkers to the headspace phase, **c**) analysis with gc-ms coupled techniques, **d**) presentation of raw data, **e**) interpretation of data using neural networks, **f**) diagnostics based on obtained molecular maps

**Fig. 2:**
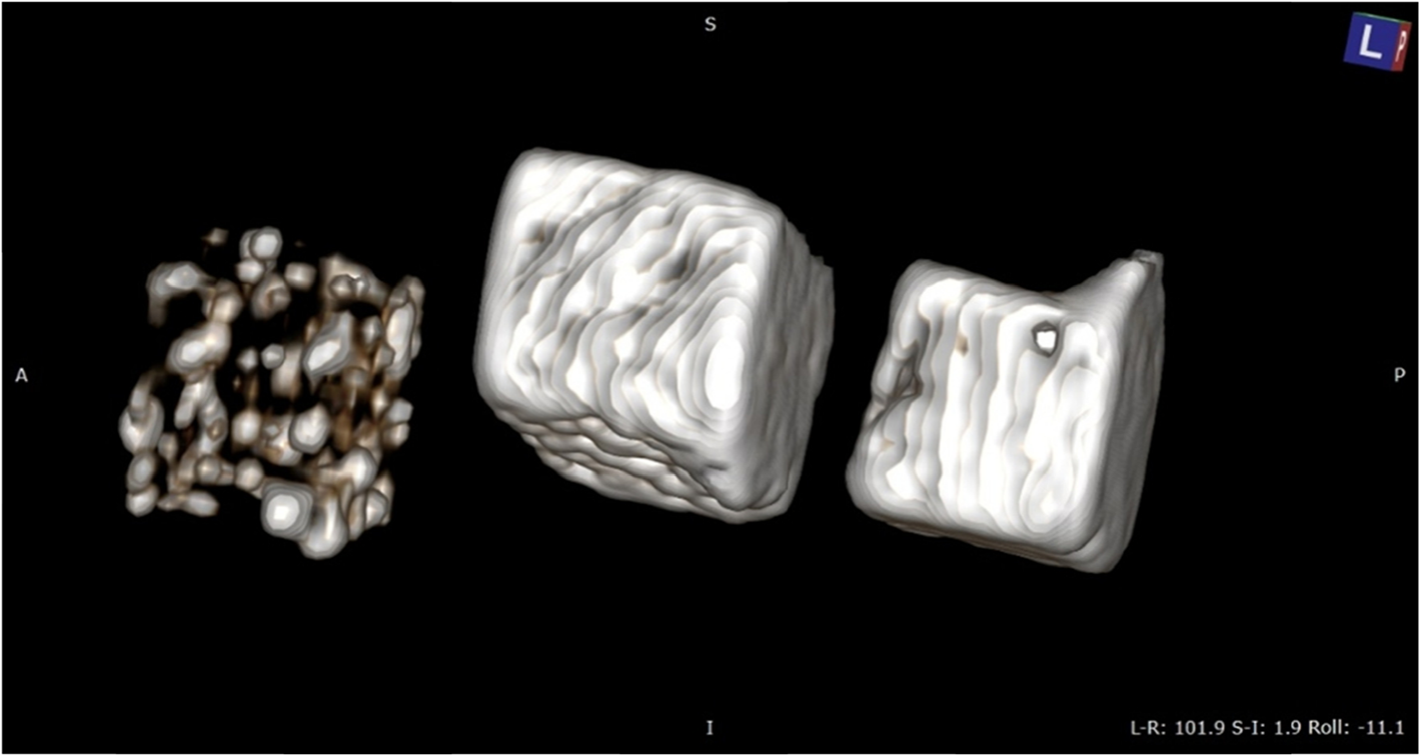
The carbon foams imaged using X-ray micro-CT (v|tome|x s, GE Sensing & Inspection Technologies, phoenix|x-ray, Wunstorf, Germany). The samples were placed on the polymer stand and scanned at 80 kV and current of 130 μA. For each carbon foam 1000 scans were obtained at a total scan time of 15 min. The established scan parameters allowed to register an image with optimal contrast and a resolution of 10 μm. The acquisition of micro-CT projections was carried out in a 8-bit grey scale in order to identify changes in the microstructure of the analysed samples. Image acquisition was carried out using the micro-CT system (GE Sensing & Inspection Technologies, Wunstorf, Germany) providing a sequence of 2D images. The reconstruction was conducted and visualizes using VGStudio MAX 2.1 software (Volume Graphics, GmbH., Heidelberg, Germany)

Respiratory phase was collected from the group of 16 swimmers before training (A), immediately after training (B) and 2 h after training (C). The breathing phase was taken using patented device PL 230578 (Fig. 1) containing a sorbent material inside (porous carbonated polyurethane, Fig. 2). Each participant breathed into the device for 2 min.

In order to discern sorption capabilities, CT scans were performed to confirm the breathing phase sorption on the surface (Fig. 1). The above illustration shows the same porous carbon foam from the left before exposure to the breathing phase, then after 10 and 20 inhalations the foam was compiled and compared. The images clearly indicate high absorption capacity of the obtained porous carbon as well as penetration of the biological material into the pores and deposition on the surface.

### GCMS analysis

The sorbent material was closed in a gas-tight container equipped with septum - a 20 ml vial. Samples were stored at a temperature below 0 °C, and then they wear prepared by agitation in 40 °C for 40 min. Analyses were performed by the use of gas chromatograph Shimadzu GCMSQP2010 Plus, equipped with a capillary column ZB5 MSi 30 m length and a diameter 0.25 mm, with film thickness 0.25 μ and installed precolumn 5 m length. Injector temperature was set to 250 °C, column temperature was changed in the range from 36 °C (1 min isothermal) to 250 °C at the rate of 8 °C/min, transfer line temperature was equal to 250 °C. As a carrier gas a helium was used. Identification of compounds was based on comparative analysis of the spectra obtained from a library of mass spectra JWS (John Wiley and Sons), and then by comparing the mass spectra and retention times of test compounds. The samples were run only once.

Numeric data is imported from text files from GC / MS (columns: retention time, absolute intensity, relative intensity). During the data import process, a preliminary verification of the correctness of the loaded data and the processing of data from the headers is carried out.

Graphs (depicting signal intensity as a function of retention time) are automatically created for all indicated samples. The analysis of charts at the initial stage of data processing will allow for verification of methods and algorithms used in analyses.

### Numerical data analysis

Our computational environment consists of: Python programming language [31], NumPy [32] numerical computation library, SciPy [33] and Matplotlib [34] libraries for data analysis and visualization of the results. We also use Jupyter Notebooks and IPython [35] for interactive analyses of the data and Scikit-learn [36] library for machine learning.

The information system will consist of several elements. It will analyze the respiratory phase signals to determine what substances are present there and what their basic characteristics are. On the basis of this data, a classifier will be created. The classifier will assign swimmers to defined groups.

Detection of substances will be performed by detecting peaks in a signal originating from GC/MS and by determining the basic features of these peaks (such as height, retention time, width, area under the graph, and others). Peaks are detected using specialized algorithms for peak detection. Determining the characteristics of the detected peaks will allow for further analysis using machine learning methods (classical machine learning as well as deep learning). Detected peaks and their characteristics will be stored in the database and will constitute input data when teaching classifiers. When creating classifiers, we will use (and test) selected machine learning and deep learning algorithms.

Peak detection in the signal is the first step in the analysis of data from the breathing phase test. Detected peaks, together with their selected features, in combination with the participant’s characteristics form the database for training the classifier.

We have analyzed several methods of peak detection. We used selected functions from Numpy numerical computation library for the Python programming language as well as our own software developed for this purpose. For example: we have tried methods based on the use of wavelet transformation (*find_peaks_cwt*) and based on the analysis of peak properties (*find_peaks*).

The method implemented in the *find_peaks_cwt* function is based on the use of a wavelet transform. It uses continuous wavelet transformation. This method was designed for finding a sharp peak in noisy data. Unfortunately, peaks that we would like to detect in the analyzed signals are not always there. In order to use this method to detect peaks of other shapes, you need to select and set the parameters carefully.

The other method, based on signal characteristics, implemented in the *find_peaks* function, proved to be the best for our purposes. The main parameters of this function are: ‘prominence’ and peak ‘width’. Using these parameters, we can limit the number of detected peaks to the number we want to analyze in the later stages of our experiments. For given parameters (prominence = 3000, width = 8), about 100–150 peaks are detected in every athlete (swimmer, patient) signal. The initial visual evaluation of the results of peak detection algorithms in the signals allows us to conclude that the appropriate parameter ranges are: prominence in the range from 3000 to 10,000 and width greater than 7.

For the detected peaks, basic characteristics such as retention time (x), absolute intensity (y), prominence, width, and peak area were calculated and recorded. They were saved to CSV files so that they could be used as a database for training the classifiers in further analyses using machine learning methods.

**Fig. 3:**
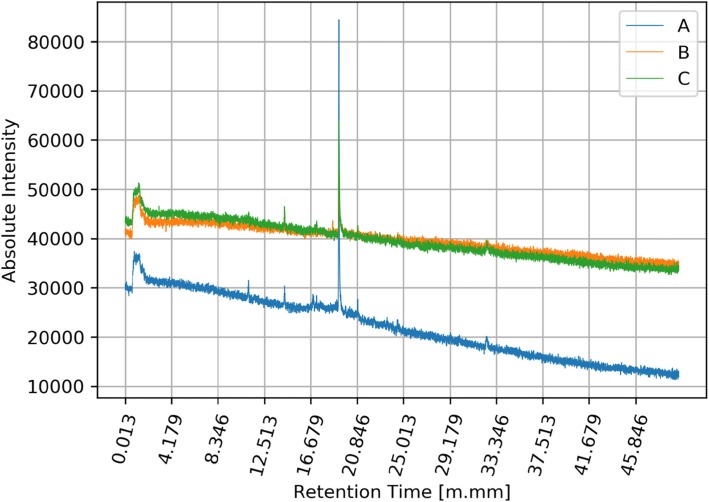
Qualitative and quantitative mean values of all signals in groups A, B and C. Y axis represents the amount in [mV] of specific biomarkers and x values represents the retention time of the collected volatile and semi-volatile compounds. Legend: blue line (A) - registered signals measured before training; orange line (B) - registered signals measured immediately after training; green line (C) - registered signals measured two hours after leaving the sports hall

**Fig. 4:**
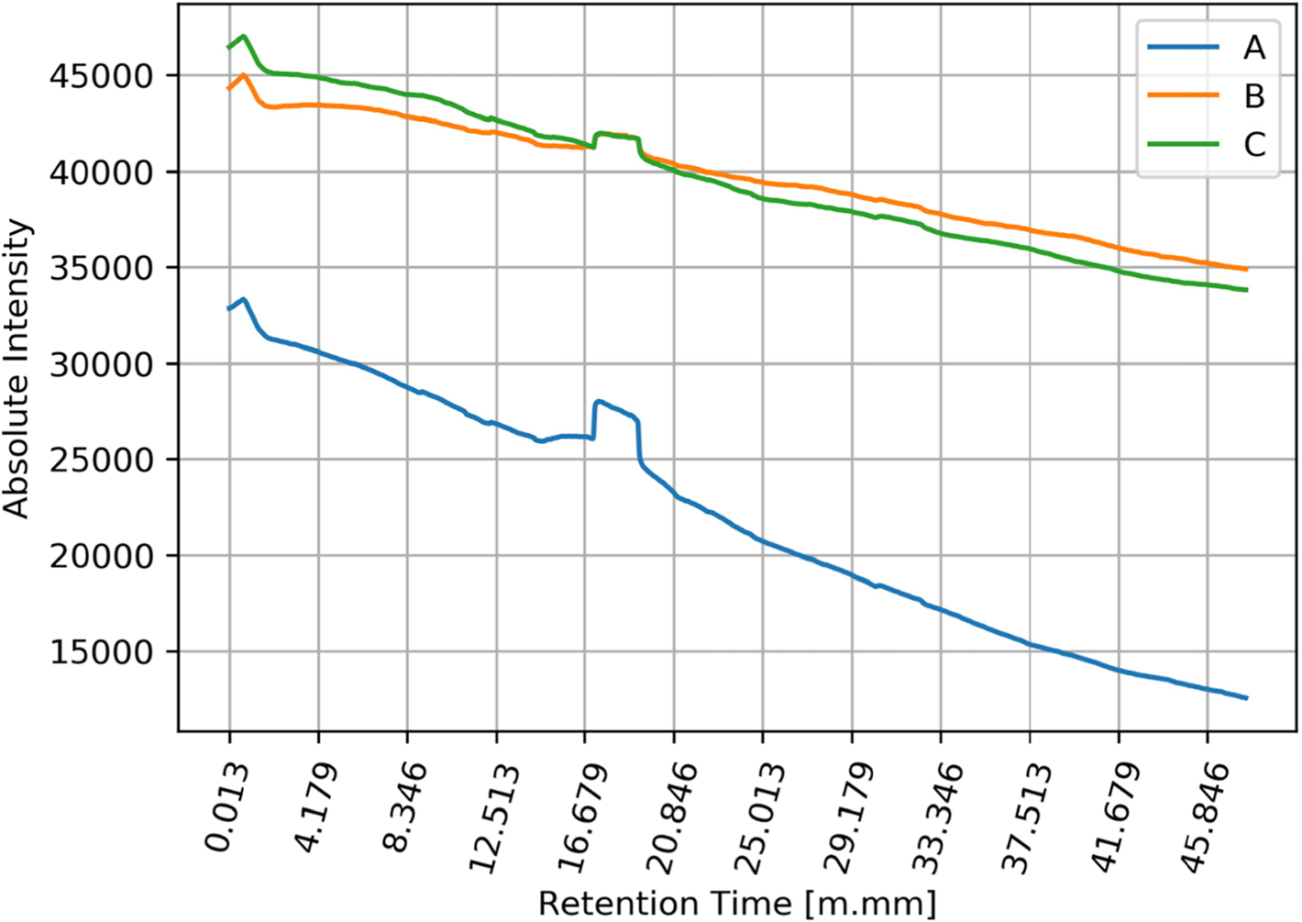
Filtered mean values of all signals in groups A, B and C. Y axis represents the amount in [mV] of specific biomarkers and x values represents the retention time of the collected volatile and semi-volatile compounds. Legend: blue line (A) - registered signals measured before training; orange line (B) - registered signals measured immediately after training; green line (C) - registered signals measured two hours after leaving the sports hall

The next stage of the analysis (but rather parallel to the previous stage) was the detection of general tendencies occurring in signals coming from different groups of signals (A, B, C). We collected a set of three signals for every swimmer. For every signal we used low-pass filtering, using the moving average method. In order to visualize the differences between signals in three groups A, B, C, we calculated the mean values ​​of filtered signals for each group. As a result, we obtained three graphs showing the average signals (intensity level) in each of the groups of signals: A, B and C. Mean raw signals are depicted in Fig. 3 and the same signals after filtration are depicted in Fig. 4.

## Results

**Fig. 5:**
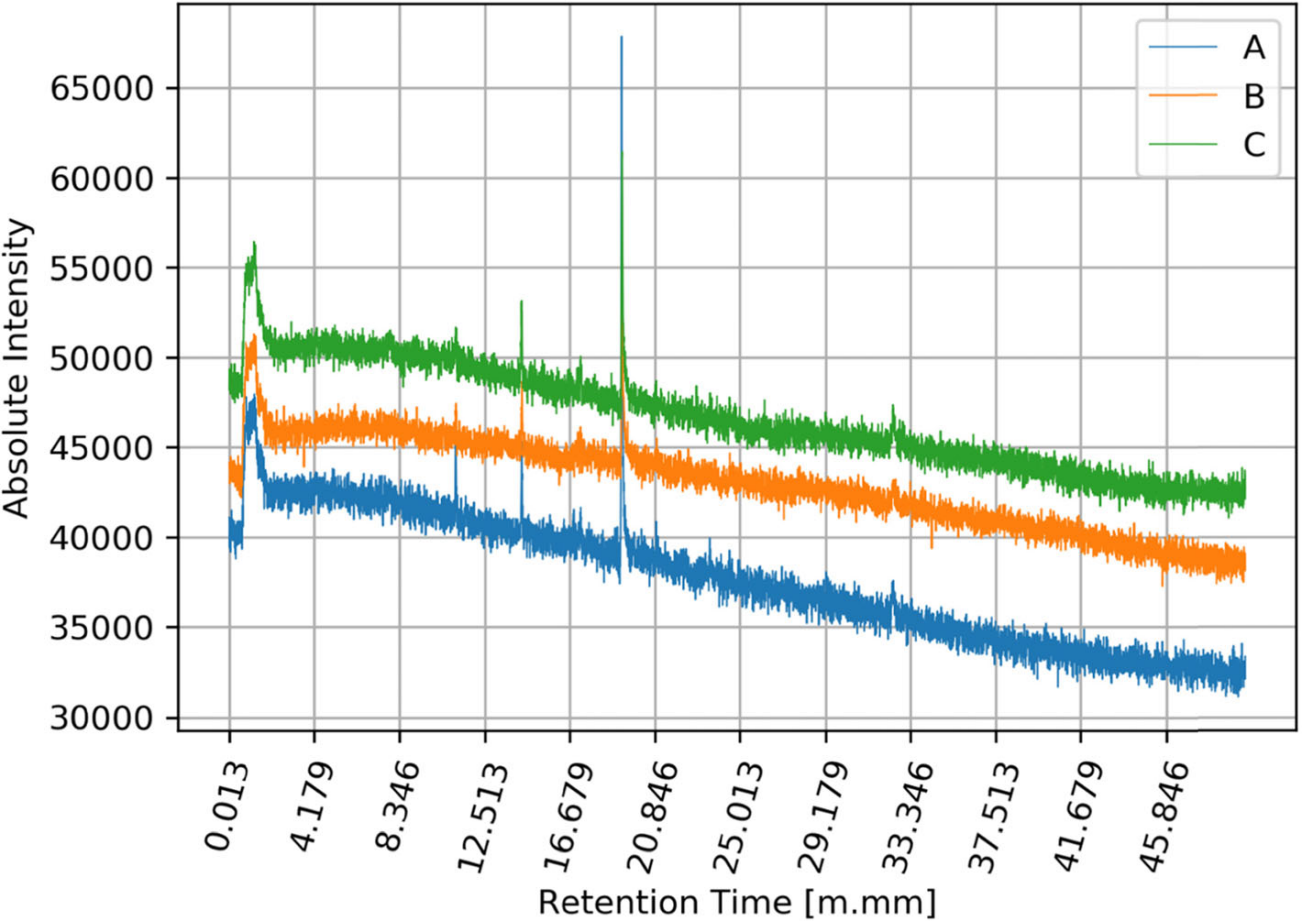
Mean values of selected signals in groups A, B and C. Y axis represents the amount in [mV] of specific biomarkers and x values represents the retention time of the collected volatile and semi-volatile compounds. Legend: blue line (A) - registered signals measured before training; orange line (B) - registered signals measured immediately after training; green line (C) - registered signals measured two hours after leaving the sports hall

**Fig. 6:**
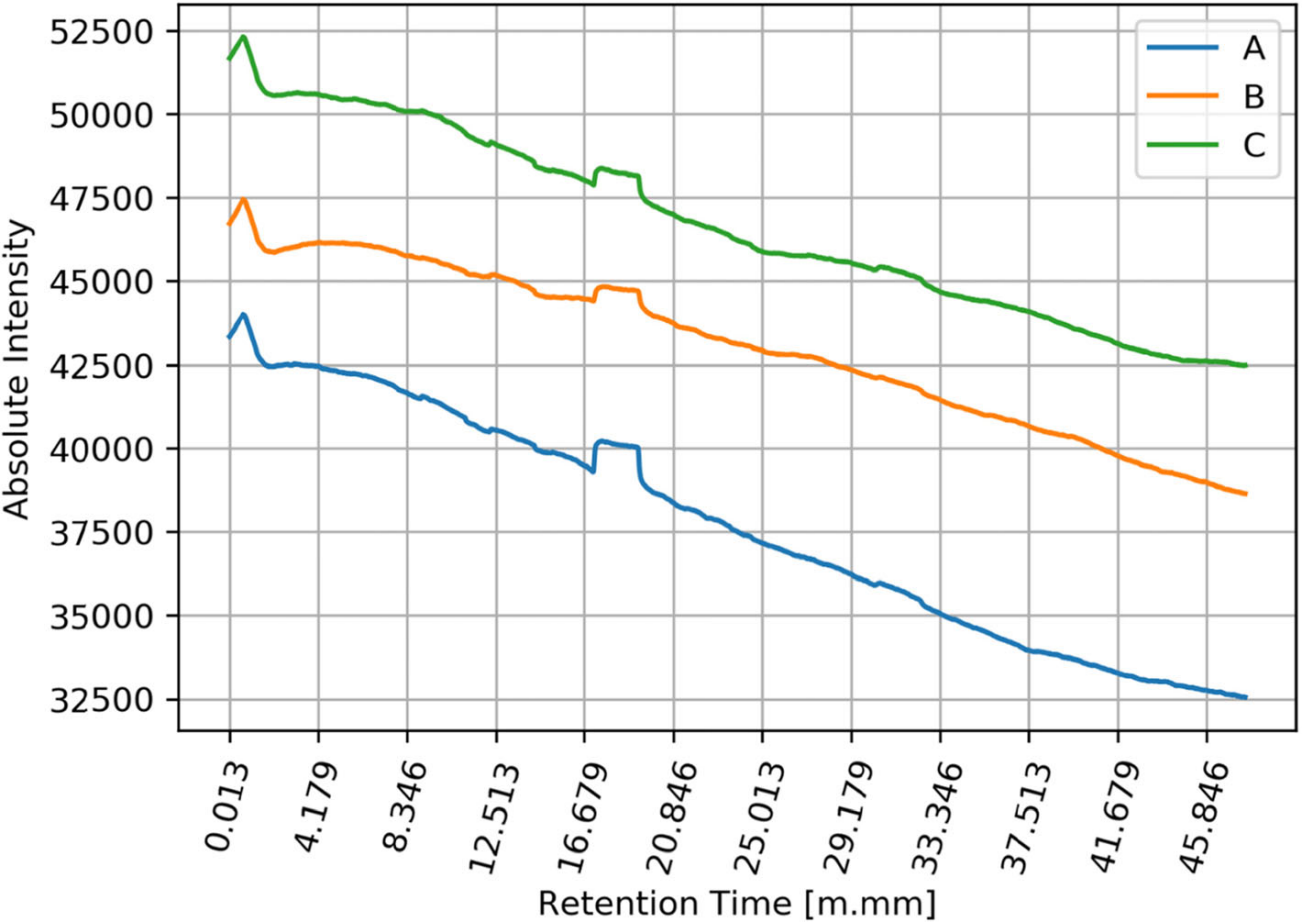
Filtered mean values of selected signals in groups A, B and C. Y axis represents the amount in [mV] of specific biomarkers and x values represents the retention time of the collected volatile and semi-volatile compounds. Legend: blue line (A) - registered signals measured before training; orange line (B) - registered signals measured immediately after training; green line (C) - registered signals measured two hours after leaving the sports hall

Many people participate in the multi-stage process of acquiring and analyzing the breathing phase. Because of that, some stages of this process are exposed to unintentional errors. In presented results, we excluded the results of unsuccessful measurements. The basis for excluding results was a low signal-to-noise ratio. Data from successful measurements were processed and depicted in Figs. 5 and 6. The image above presents moving averages of mean signals calculated for three groups of swimmers A, B, C. Figure 6 shows that there are differences between levels of filtered average signals for different groups.

What explains these different levels? Each line in the figure is related to the average level of substances found in the breathing phase. From the presented graphs we can assume that in each group A, B and C, the amount of substances present in the breathing phase are different. The distances between the lines suggest significant differences between the three groups of results.

During the experiment, athletes dealing with professional swimming were examined regarding the composition of exhaled air before training (on empty stomach/ fasting) after training and two hours after leaving the swimming pool hall. Results showed clear changes between the registered signals for group A pre-training, group B immediately after training and group C, measured two hours after leaving the sports hall. The results clearly indicate that during the training there was an increase in the content, quantitatively and qualitatively, of volatile organic compounds in exhaled air in swimmers. Explanation of this phenomenon may rely on the reaction of chlorine compounds taken into the bioreactor – which are the lungs –during swimming and its reaction with the gas content of the lungs and airways, including hydrocarbons derived from the gastrointestinal tract.

The human respiratory system is made up of lungs and respiratory tract, the operated air travels to the alveoli and back. Respiratory include nostrils, nasal cavity, throat, larynx and trachea, which branches into two bronchi - left and right, leading to the lungs. The pulmonary vesicles are surrounded by a dense network of capillary blood vessels. Oxygen and carbon dioxide exchange between the blood and air in the alveoli occurs through the walls of the alveoli. Gas exchange occurs by diffusion. Under normal physiological conditions, alveolar air contains a large amount of volatile organic substances diffusing from the blood, through the pulmonary alveolar membrane, according to a vapor pressure gradient. In addition, in exhaled air, substances belonging to various chemical classes: saturated hydrocarbons (methane, ethane, pentane), unsaturated hydrocarbons (isoprene), aromatic hydrocarbons (benzene), aldehydes (ethanal, methanal, acetaldehyde), ketones (acetone), alkoxy (methanol, ethanol, 2-propanol), esters (methyl acetate, ethyl acetate), sulfur compounds (methanothiol, ethanethiol, carbon disulfide, carbonyl sulfide, hydrogen sulfide, dimethyl sulfide), nitrogen-containing compounds (dimethylamine (DMA), trimethylamine (TMA), ammonia) and others [37]. The breath sample may contain several sets of volatile organic compounds collected at nmol levels [38–40].

Exhaled air, in addition to organic groups, also contains a number of inorganic substances, such as carbon monoxide, nitrogen oxides, and ammonia. In addition, non-volatile organic compounds such as leukotrienes, cytokines, prostoglandins, isoprostanes and hydrogen peroxide derived from metabolism are also observed in the exhaled air [41]. These endogenous derivatives of cellular metabolism occur in the form of an aerosol which, after freezing, forms respiratory condensate [42, 43].

The formation of dichloromethane or chloroform from chlorine with methane, may significantly affect the metabolic changes in breath, but it is assumed that in the same chemical process, first and second order amines are formed and they can act as a shield and bronchial distally on the lungs of athletes. Detailed analysis of the full composition of volatile organic compounds and changes under the environmental impact of the indoor swimming pool, especially chlorine and chlorine compounds will be the basis of the next work. Results shows a clear response of the organism of a swimmer to chlorine compounds based on changes in the characteristics of the signal recorded before and two hours after training, these changes indicate a significant effect of air composition on the indoor swimming pool on metabolism and lung function in athletes. Accurate assessment of quantitative and qualitative changes in biochemistry will allow proper training as well as maintaining the content of volatile chlorine compounds derived from the disinfection of swimming pool water for the health and sports results of swimmers. It is necessary to control the swimmer’s time in the indoor swimming pool, as well as the free chlorine and chlorine bound in water. However, in full analysis of the process of releasing chlorine compounds into the air of the indoor swimming pool, it is necessary to control the composition of exhaled air in both athletes and all personnel of the indoor swimming pool and to control their exposure to chlorine derivatives.

## Discussion

According to our best knowledge we are the first ones to assess the exhaled gas analysis on the highly porous aseptic material and further assessed with the use of GC/MS in competitive swimmers which are strongly exposed to low dosage chlorine compounds in the breathing air.

Considering that millions of people are exposed to chlorine atmosphere our data seems to be of high clinical significance.

Sport swimming was a discipline in which the right swimming technique and “feeling for the water” developed during training in the water environment guaranteed effective movement in water and successful competition in sport. Even a few days of absenteeism in training caused by the harmful effects of chlorine on the swimmer’s body would cause disorders in neuromuscular coordination. Compensation of training deficiencies in water through the implementation of training programs on land could also adversely affect the ability to move quickly in the water. Resistance training affected changes in bone density and connective tissue [44, 45]. There was no doubt that exercises with the use of retaining rubbers, free weights and different types of trainers not only increased the strength of the main muscles causing motive motion, but also contributed to muscle hypertrophy. It has been supported that large muscle hypertrophy and reduced flexibility could cause increased resistance in the water, which would negatively affect the performance of swimming [46]. Therefore, resistance training on land could only be conducted in conjunction with training in water, because due to the enormous amount of training in a natural environment for swimmers and a very large number of endurance exercises, it was unlikely that significant hypertrophy occurred [47, 48]. Training in the open water environment is limited in many countries by the seasonal climate changes and difficulties in relapse in the swimming pool in open waters.

The mentioned influence of chlorine and its volatile compounds was clearly visible during the research. As a result, a clear increase in baseline in specific range was observed in all athletes after exposure to factors in the atmosphere of the indoor swimming pool for the duration of the workout. The increase in baseline value was due to the increased presence of volatile compounds from metabolic processes. Such a result indicates that the content of volatile chlorine derivatives and other gases as well as organic and inorganic substances contained in the indoor pool air has a significant impact on the composition of the respiratory phase of the trainees. The respiratory phase, however, has a significant impact on the correct supply of oxygen to muscles and the removal of carbon dioxide, a change in its composition can cause not only metabolic changes but also adversely affect the athletes’ shape due to the presence and formation of chloroamines in contact with the gases of the digestive process. As a result of feedback synergy, the body is automatically protected and the negative effects of chlorine and outlying dichloromethane on the upper and lower respiratory tract are reduced.

The proposed method of chlorine retention in the airways seemed to be a practical tool for determining the chlorine concentration threshold in the air. Above this threshold, it will be recommended to use more effective ventilation or additional substances that bound volatile chlorine compounds. In addition, this method will allow biochemical assessment of the correctness of the ventilation. This type of testing should be routinely carried out in professional swimmers to maintain optimal lung performance as well as prevent various diseases associated with the carcinogenic effects of chlorine compounds such as trichloromethane.

After analyzing a number of samples from swimmers, it would be possible to develop a base of substances most often found in the breathing phase under investigation. Thanks to this database, after receiving the sample, the system would automatically provide which substances have been identified (and with what probability). The most important feature of the developed information system was that it would enable automatic processing and analysis of data from breathing phase samples.

The system was created in the Python programming language environment (Python ecosystem). Python is an interpreted high-level language for general-purpose programming. It has been widely used in data analysis and in the field of modern machine learning. In addition to the extensive standard library, there were many additional very good libraries available on the market (e.g. for statistical analysis, machine learning or deep learning methods). In the future, ultimately, the system would be available in the form of a web application, so one could use it through a simple web browser (such as Internet Explorer, Firefox or Safari). This system architecture would allow avoiding the cumbersome process of installing the software along with the appropriate libraries.

### Study limitations

The present work is the first stage of research, the purpose of which was to obtain information on whether there are changes in the composition of the air exhaled by swimmers, caused by swimming training. Studies have confirmed that the breathing phase varies depending on the duration of exposure to the agent, which is chlorine, but at this stage it is not possible to indicate other volatile compounds that may have formed in the respiratory tract, and which the measuring apparatus has signaled. This will be the subject of our research in the near future.

Although presented data shows a novel approach towards chronic reaction to persistent chlorine exposition it has several limitations which should be addressed.

Firstly, the data analysis was performed in male subject only. We are aware that there are huge differences in asthma related symptoms and airway reactivity when comparing male and female population [49]. Including males and females was considered at the stage of study protocol preparation, however this study was not addressed to asthma but to airway response of healthy airways. Therefore, taking into account changing airway response and immunity [49] and possible changes in the female airway immunity caused by genetic factors [50] and possible changes in females airway reactivity and immunological response in different menstrual phases we have decided not to include females into study protocol [51]. However, considering obtained strong signal form male subjects we have decided to assess in the future response of female respiratory system to chlorine compounds low level exposition.

Secondly, we are aware that our sample size was rather small however it has to be underlined that most of the studies performed on patients highly exposed into chlorine gas were performed on small patients groups and usually after acute exposition [19], even if the observed consequences were long lasting [52]. Small study population which should be considered as study limitation ad the end turned out not to limit our findings as we have found that respiratory system response to low dose chlorine exposure was clinically significant and whereas our method proved to be clinically sensitive in detecting this response, we cannot consider small study population as a real study limitation.

### Future research areas

Based on our early findings it may be concluded that our study should be carried on female competitive swimmers’ population preferably in both phases of menstrual cycle. If this reaction is similar in men and women, it is worth carrying out research into different age groups in the future. Probably the organism’s response depends on the athlete age [53].

Taking into account that competitive swimmers represent a small population and that chlorine particles are accepted as highly reactant with accepted dose response [54] it should be speculated that in expositions such as indoor swimming similar mechanism should be considered. Therefore, larger studies with the use of our method are required to assess the respiratory system response not only in competitive swimmers but in other populations such as lifeguards (long exposition with relatively small mean minute ventilation), but are subject to repeated exposition [55] which as well as in occasional swimming pools users (different age groups including children of subjects, different potential cigarette smoke exposure or with presence of chronic respiratory systems such as rhinitis, asthma, chronic obstructive pulmonary disease or others).

## Conclusions

The conducted studies showed the existence of significant differences between the registered signals before training, immediately after training and 2 h after training. The obtained results clearly indicate an increase in the content, quantitatively and qualitatively volatile organic compounds in the breath measured during training. By analyzing the compounds that appear in the exhaled air and their accurate evaluation, it will be possible to develop new methods for training swimmers, taking into account, for example, the maximum time the swimmer can stay in the indoor pool. The research will also allow controlling the exposure to harmful chlorine derivatives, which will contribute to eliminating the negative effects associated with the use of the pool. The presented graphs clearly show changes in the metabolic profile before and after exposure to indoor swimming pool conditions. The graphs show the increase in baseline and the intensity of signals from semi volatile compounds between 16:00 and 19:00 min retention time, additionally. Additionally, a change in the trend of the curve was observed before and after exposure. Worth mentioning is the increase in the intensity of hardly volatile compounds present in the range over 30 min retention time which indicates a change in the full metabolic profile in the semi-volatile and hardly volatile chemical compounds present in the breath. This difference is smaller but also noticeable in the range of readily volatile compounds between zero- and fourth-minute retention time, in this regard, are observed all derivatives of oxides and sulfides, including carbon oxides and nitrogen. The results clearly indicate the need for continuous monitoring of the surface layer due to its significant influence on the composition of the exhalation phase in people exposed to the atmosphere of the indoor swimming pool in which chlorine and chlorine compounds were used as a disinfectant compound.

## Data Availability

Data sharing requests from appropriate researchers and entities will be considered on a case-by-case basis. Interested parties should contact the corresponding author.
